# Quality improvement approach for surgical-site infection prevention in a Philippine provincial hospital

**DOI:** 10.1017/ash.2023.368

**Published:** 2023-09-29

**Authors:** Anthony Abustan, Unarose Hogan, Julie Winn, Paul Pagaran, Joan Littlefield, Ted Miles

## Abstract

**Background:** Globally, the 30-day cumulative incidence of surgical-site infections (SSI) was 11% (95% CI, 10%–13%) based on the systematic review and meta-analysis derived from 57 studies. SSIs are poorly studied in the Philippines. Americares and its hospital partner, Camarines Norte Provincial Hospital, Philippines, sought to reduce SSIs through (1) establishing SSI surveillance in the hospitals’ surgical departments, (2) implementing quality improvement processes, and (3) developing and implementing an SSI prevention care bundle. **Methods:** A quality improvement methodology was used to introduce SSI surveillance and care-bundle checklist in partnership with Americares. Using paired *t* tests, pre- and posttest scores of the SSI care bundle training were analyzed. SSI surveillance was established based on the adapted CDC criteria. All clean surgeries were monitored except orthopedic surgeries. The number of surgeries performed, monitored, and SSIs identified were documented using the surveillance forms and plotted using Microsoft Excel software. A care bundle based on WHO evidence-based interventions for SSI prevention was designed and implemented. Compliance with the SSI care bundle was documented using Microsoft Excel. The relationship between the use of a care bundle and SSIs was analyzed using the Pearson correlation coefficient. **Results:** An online SSI care bundle training session was conducted. Overall, 150 participants had a mean pretraining test score of +6.46. After the training was conducted, the same participants had a mean posttraining test score of + 1.76). a statistically significant increase of 5.29 (95% CI). Thereby, the mean score difference after training showed that knowledge increased overall. These findings show an average of 90.43% compliance with the SSI care-bundle checklist over the 18-month window from May 2021 to November 2022. From a baseline of 0%, compliance increased from 80% upon its introduction in May 2021. Lastly, the SSI incidence rate from May 2021to November 2022 averaged 1.89%. The days between reported SSIs averaged 16.85. No baseline was available for comparison prior to the introduction of the surveillance and care bundle. A Pearson *r* data analysis (n = 1,850) was used to determine the relationship between the use of the care bundle and SSIs. The data illustrated a moderate negative correlation (*r* = −.31). Therefore, higher care-bundle compliance yielded fewer SSI cases. **Conclusions:** The use of an evidence-based care bundle paired with a local quality improvement process significantly improved SSI prevention and surveillance. Future studies are needed that include clean-contaminated, contaminated, and dirty surgical cases to test the degree of SSI reduction possible.

**Disclosures:** None

